# Development and Validation of a Risk Prediction Model to Identify Women With Chronic Obstructive Pulmonary Disease for Proactive Palliative Care

**DOI:** 10.1111/resp.70005

**Published:** 2025-02-16

**Authors:** Begashaw Melaku Gebresillassie, John Attia, Dominic Cavenagh, Melissa L. Harris

**Affiliations:** ^1^ School of Medicine and Public Health The University of Newcastle Newcastle New South Wales Australia; ^2^ Centre for Women's Health Research University of Newcastle Newcastle Australia; ^3^ Hunter Medical Research Institute Newcastle New South Wales Australia

**Keywords:** COPD, mortality, palliative care, prediction model

## Abstract

**Background and Objective:**

Proactive palliative interventions can improve symptom control and quality of life in individuals with chronic obstructive pulmonary disease (COPD); however, they are often underutilised. This study aimed to develop and validate a prediction model to identify women with COPD in their last year of life to facilitate timely palliative care referrals and interventions.

**Methods:**

Data from 1236 women diagnosed with COPD from the 1921–1926 Australian Longitudinal Study on Women's Health cohort, linked to administrative health records, were analysed. We employed Lasso regression and multivariable logistic regression to select predictors. To assess the predictive performance of the model, we used the area under the receiver operating characteristic (AUROC) curve, calibration plot, and calibration metrics. The Youden index was used to establish the optimal cutoff point for risk classification. The clinical utility of the model was evaluated using decision curve analysis (DCA).

**Results:**

The final model to predict 1‐year all‐cause mortality included six predictors: smoking status, body mass index, needing regular assistance with daily activities, number of supplied medications, duration of illness, and number of hospital admissions. The model performed well, with AUROC of 0.82 (95% CI: 0.80–0.85) and showed excellent calibration. Using a cutoff of 56.6% predicted risk, the model achieved a sensitivity of 72.3%, specificity of 77.7%, and accuracy of 75.0%. The DCA indicated that the model provided a greater net benefit for clinical decision‐making.

**Conclusion:**

Our prediction model for identifying women with COPD who may benefit from palliative care has shown robust predictive performance and can be easily applied, but requires external validation.

## Abstract 2

We developed a predictive model to identify older women with COPD who are at a high risk of 1‐year mortality. The model, based on six readily available, non‐invasive, and inexpensive predictors, demonstrated excellent predictive accuracy. This can aid in identifying high‐risk patients for timely palliative care interventions, potentially improving outcomes.

**See related** article

**Summary**

We developed a predictive model to identify older women with COPD who are at a high risk of 1‐year mortality. The model, based on six readily available, non‐invasive, and inexpensive predictors, demonstrated excellent predictive accuracy. This can aid in identifying high‐risk patients for timely palliative care interventions, potentially improving outcomes.

## 1 Introduction

Chronic obstructive pulmonary disease (COPD) is a complex condition characterised by persistent respiratory symptoms, limited airflow, and increased inflammation of the airways and lungs [1]. Globally, COPD is a major contributor to premature mortality, ranking third in 2010, and is projected to be the fourth leading cause of death and fifth in disease burden by 2030 [1, 2, 3]. In Australia, COPD is particularly prevalent among older women, with a study indicating a prevalence of 7.5% in individuals aged 40 years and older, rising to 29.2% in those aged 75 years and older [4]. COPD remains one of the leading causes of death and disability among older women in Australia, with a 1‐year mortality rate of 20.7% reported in individuals with COPD receiving long‐term oxygen therapy [5].

Evidence suggests that women are more susceptible to smoking and airborne contaminants, leading to distinct clinical presentations [6, 7]. Women with COPD often report more pronounced dyspnoea, higher rates of anxiety and depression, undernutrition, and comorbid conditions such as lung cancer and osteoporosis [7, 8]. These factors, coupled with a more significant impact on quality of life, highlight the importance of understanding the gender‐specific impact of COPD [6, 7, 8]. As the disease advances, women may develop symptoms characterised by a progressive decline in overall function, loss of independence, and reduction in health‐related quality of life [9]. The final years are often marked by progressive functional decline, frequent exacerbations, poor health‐related quality of life, and increasing dependency on caregivers and the healthcare system [10]. In particular, three‐quarters of patients are dependent on caregivers, and around half are housebound [11, 12, 13]. Early palliative care interventions have demonstrated significant benefits, including improved symptom control, improved quality of life, increased patient comfort, and reduced hospital admissions for individuals with COPD [14, 15, 16]. However, these interventions remain underutilised and are not part of routine care [14, 15, 17].

Despite the benefits of palliative care for individuals with advanced COPD, timely referrals are limited due to prognostic uncertainty and challenges in determining the optimal referral timing [15, 18]. Ideally, palliative care interventions, including advanced care planning, should be based on patient needs such as unaddressed symptoms, psychosocial suffering, or low quality of life, as well as patient prognosis [18]. However, a lack of reliable prognostic estimates complicates this process [18]. A prognostic tool that provides precise mortality risk estimates could effectively complement a needs‐based approach, thereby enhancing the confidence of healthcare providers' and caregivers' and reducing hesitation in making timely referrals [18].

Current approaches to identifying patients in need of palliative care rely heavily on clinical judgement, as well as claims data; however, both approaches have limitations [18, 19]. The available survival prediction tools for palliative care applications, such as the Palliative Performance Scale [20] and Palliative Prognostic Score [21], are based on expert‐curated predictors, such as functional ability and oral intake. More specifically, the available models for individuals with COPD have been limited to specific clinical settings, including outpatients, inpatients, and stable patient populations with controlled symptoms and no recent acute exacerbations [22, 23], and they mainly focus on long‐term mortality predictions [23, 24]. Furthermore, these models have methodological limitations, including inadequate sample sizes, improper handling of continuous and categorical variables, failure to evaluate or disclose relevant model performance metrics, lack of consideration for potential overfitting and optimistic bias in model performance, and lack of internal and external validation [22, 24].

The development and validation of new reliable prediction tools could assist healthcare providers in the early identification of high‐risk individuals with COPD who may benefit from timely palliative care interventions. Timely palliative care has the potential to meet patient needs and improve patient satisfaction as well as healthcare providers and families [16, 17]. Therefore, this study aimed to develop and validate a multivariable prediction model to predict 1‐year all‐cause mortality in women with COPD from the 1921–1926 Australian Longitudinal Study on Women's Health (ALSWH) cohort.

## 2 Methods

### 2.1 Study Design

Data were obtained from the 1921–1926 cohort of the ALSWH linked to state‐based administrative data. ALSWH is an ongoing, nationally representative, population‐based cohort study [25]. In 1996, women aged 70–75 years were randomly selected from the National Medicare Health Insurance database, which included all Australian citizens and permanent residents. The ALSWH sample of older women was generally representative of Australian women of the same age but included a greater proportion of women who were married or living with their partner, as well as a greater proportion of women with post‐school qualifications, compared to the 1996 Australian Census [25, 26]. A total of 12,432 women who responded to the first survey in 1996 completed surveys every 3 years until survey 6 (2011) and thereafter mini‐surveys on a 6‐month rolling basis. The details of the study design are available elsewhere [26, 27].

### 2.2 Data Sources

This study utilised data from six ALSWH surveys (1996–2011), linked to: (i) Admitted Patients Data Collection (APDC) and Emergency Department Data Collection (EDDC) which provide data on hospital service utilisation, including public and private hospital admissions from individual state and territory‐based datasets (except for Australian Capital Territory, Northern Territory, South Australia, and Tasmania, which only include public hospital admissions); APDC and EDDC include details on the number of hospital admissions, admission and separation dates, procedures performed, and related diagnoses. (ii) ALSWH‐developed Common Conditions from Multiple Sources (CCMS) datasets that provide information on the diagnoses of common conditions such as asthma, COPD, dementia, and diabetes, including sources and the corresponding dates (details about the data sources and disease‐related algorithms can be found in the Supporting Information). (iii) National Death Index (NDI), which captures all deaths in Australia, using probabilistic matching to identify deaths among ALSWH participants based on name, date of birth, and gender.

### 2.3 Participants

Participants for this analysis were selected from the ALSWH 1921–1926 cohort, who were diagnosed with COPD until 2021. Of the 12,432 women who completed the first survey in 1996, 11,351 consented to data linkage and were eligible for the analysis. Women were excluded from the analysis if they did not have COPD, resided in the Australian Capital Territory and Tasmania (due to incomplete APDC and EDDC data before 2003), or died before 2003. Further exclusions were made for those with incomplete hospital and survey data.

**FIGURE 1:**
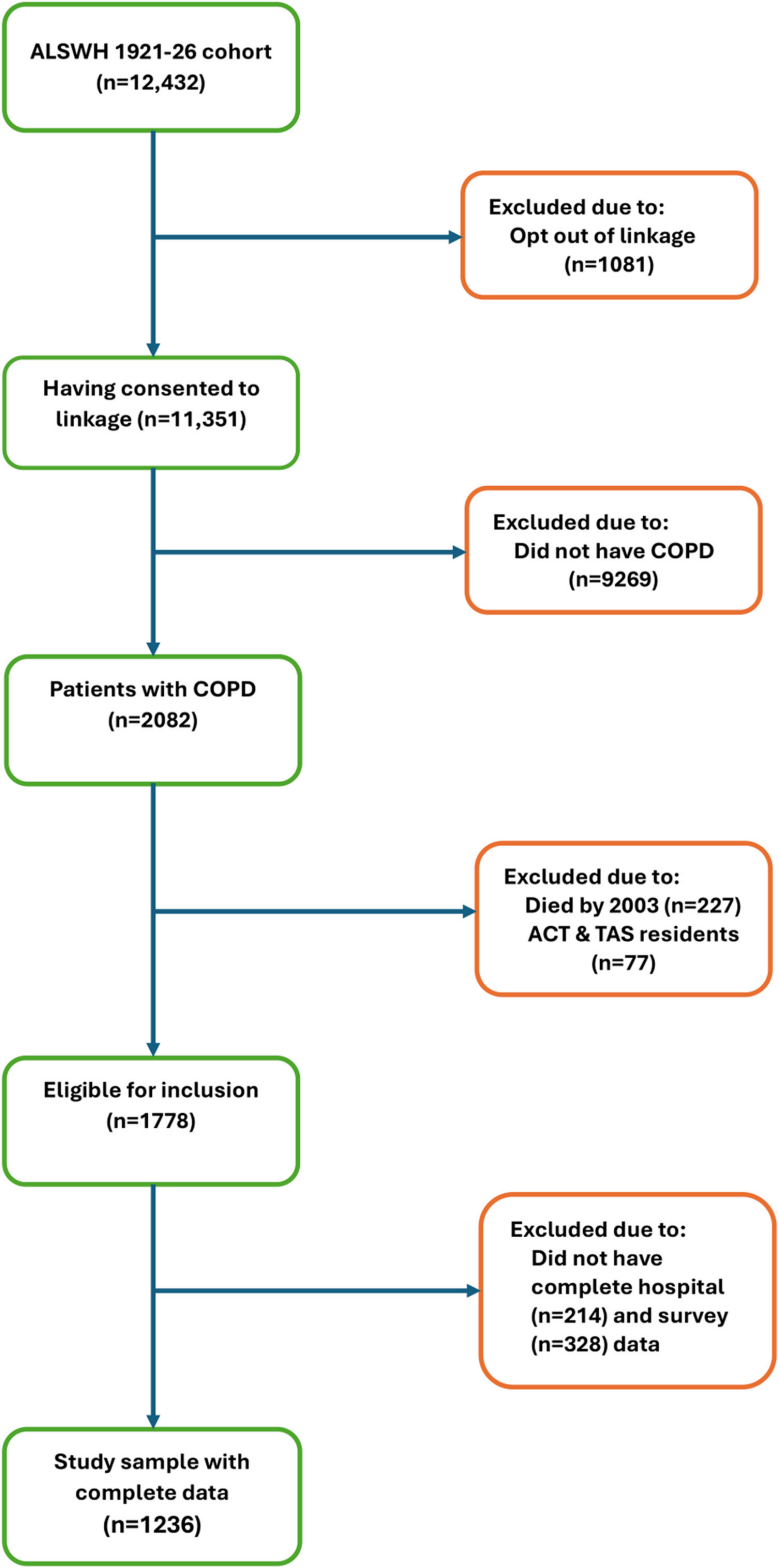
Flow diagram depicting the study sample included in the analysis.

Cases were defined as women with COPD who died between 2003 and 2013. This timeframe was chosen because complete APDC and EDDC data were available from 2003 onwards, and the last full survey data available was from 2011. For each case, a control was selected randomly on a 1:1 basis by looking back 1 year from the date of death of the case to find a woman who was at risk of death at the 1‐year mark. Controls were not selected more than once throughout the study period, and they were not reclassified as cases, even if they died later (Figure 1).

### 2.4 Measures

#### 2.4.1 COPD Case Ascertainment

COPD was ascertained using the ALSWH‐developed CCMS datasets, which involved the use of multiple state‐based and national data sources such as the ALSWH survey, APDC, EDDC, NDI, Pharmaceutical Benefits Scheme, and aged care assessments [28]. Full details are provided in the Supporting Information.

#### 2.4.2 Predictors

A set of socio‐demographic, behavioural, and clinical characteristics, was considered as potential candidate predictors for model development. These predictors were selected a priori based on our systematic review [24], their statistical strength of association with the outcome variable, and clinical relevance (Table S1 in the Supporting Information).

#### 2.4.3 Outcome

The main outcome was 1‐year all‐cause mortality obtained from the NDI [25].

#### 2.4.4 Data Processing and Analysis

The process of model development and evaluation, as well as the reporting of results, adhered to the methodological standards outlined by the Transparent Reporting of a multivariable prediction model for Individual Prognosis Or Diagnosis (TRIPOD) guidelines [29]. Predictor data for cases were collected from the most recent survey of each patient that was closest to the 1‐year mark prior to their death, whereas data for controls were obtained from the most recent survey using matched cases' death date. Missing data for time‐invariant predictors were imputed from previous survey waves where appropriate.

Hospital admission and length of hospital stay were obtained from APDC for the 12 months before the last year of life for cases and calculated for controls based on the matched cases' death date. The assumptions of logistic regression, including checking for linearity in continuous predictors and multicollinearity, were undertaken. Owing to a violated linearity assumption and significant right skewness, the number of hospital admissions was log‐transformed. The data were cleaned using STATA‐17, and all analyses were conducted using R version 4.4.0.

#### 2.4.5 Model Development

Univariate logistic regression was initially used to explore the association between each potential predictor and 1‐year all‐cause mortality. Variables with a *p* value < 0.25 in univariate analysis were included in the Lasso regression. Lasso regression was then applied to select relevant predictors. Subsequently, a multivariate logistic regression model was applied to refine the final model by incorporating the results of the Lasso regression model and the aforementioned selection criteria.

#### 2.4.6 Model Performance Evaluation

Model discrimination was assessed by calculating performance metrics such as the area under the receiver‐operating characteristic curve (AUROC), sensitivity, and specificity. Model calibration was assessed using calibration metrics and calibration plots of predicted and observed probabilities. Decision curve analysis was conducted to explore the net benefits of the model. Additionally, internal validation of the developed model was assessed using a bootstrapping technique; 10,000 bootstrap iterations were performed to assess the discrimination and calibration performance of the model.

## 3 Results

### 3.1 Baseline Characteristics

A total of 1236 individuals (*n* = 618 cases and *n* = 618 controls) from the 1921–1926 cohort with COPD were included in this analysis. The mean baseline age in 1996 was 72.4 ± 1.5 years. More than half of the participants lived in regional or remote areas (*n* = 655, 53%), and a significant majority were non‐partnered (*n* = 876, 70.9%). More than half of the participants were current or past smokers (*n* = 699, 56.6%), with a higher prevalence among cases (64.7% vs. 48.4%). Alcohol consumption was similar between the two groups (57.3% vs. 56.8%). Physical limitations to performing daily tasks were prevalent among the study participants, with 71.1% having difficulty climbing stairs and 54.6% having difficulty walking 100 m. Most importantly, nearly one‐third of the participants had difficulty bathing or dressing themselves (*n* = 404, 32.7%) and regularly needed help with daily tasks due to limitations caused by long‐term illness, disability, or frailty (*n* = 358, 29.0%). Most participants had comorbid chronic conditions such as hypertension (*n* = 1095, 88.6%), ischaemic heart disease (*n* = 862, 69.7%), and diabetes mellitus (*n* = 309, 25.0%). Additionally, the majority of participants had been supplied at least one medication during the past 4 weeks (*n* = 1143, 92.5%) (Table 1).

**TABLE 1:** Socio‐demographic, behavioural and health characteristics of women with COPD from the 1921–1926 ALSWH cohort to predict 1‐year all‐cause mortality (*n* = 1236).

Characteristics		Died^a^ *N* = 618	Alive^a^ *N* = 618	Total^a^ *N* = 1236
Baseline age	Mean ± SD	72.6 ± 1.5	72.2 ± 1.4	72.4 ± 1.5
Area of residence	Major cities	285 (46.1%)	296 (47.9%)	581 (47.0%)
	Regional	326 (52.8%)	313 (50.6%)	639 (51.7%)
	Remote	7 (1.1%)	9 (1.5%)	15 (1.3%)
Marital status	Partnered	205 (33.2%)	155 (25.1%)	360 (29.1%)
	Non‐partnered	413 (66.8%)	463 (74.9%)	876 (70.9%)
Smoking status	Never smoked	218 (35.3%)	319 (51.6%)	537 (43.4%)
	Current/ex‐smoker	400 (64.7%)	299 (48.4%)	699 (56.6%)
Alcohol consumption	Non‐drinker	264 (42.7%)	267 (43.2%)	531 (43.0%)
	Drinker	354 (57.3%)	351 (56.8%)	705 (57.0%)
DSSI Social Interaction sub score^b^	Mean ± SD	8.5 ± 1.6	8.6 ± 1.6	8.6 ± 1.6
Body mass index in kg/m^2^	Underweight	224 (36.3%)	157 (25.4%)	381 (30.8%)
	Healthy weight	212 (34.3%)	266 (43.0%)	478 (38.7%)
	Overweight/obese	182 (29.5%)	195 (31.6%)	377 (30.5%)
Private health insurance	Yes	202 (32.7%)	234 (37.9%)	436 (35.3%)
	No	416 (67.3%)	384 (62.1%)	800 (64.7%)
Compared to 1 year ago, health now	Better	54 (8.7%)	71 (11.5%)	125 (10.1%)
	Same/worse	564 (91.3%)	547 (88.5%)	1115 (89.9%)
Assistance with daily tasks	Yes	233 (37.7%)	125 (20.2%)	358 (29.0%)
	No	385 (62.3%)	493 (79.8%)	878 (71.0%)
Difficulty climbing one flight of stairs	Yes	467 (75.6%)	412 (66.7%)	879 (71.1%)
	No	151 (24.4%)	206 (33.3%)	357 (28.9%)
Difficulty walking 100 m	Yes	379 (61.3%)	296 (47.9%)	675 (54.6%)
	No	239 (38.7%)	322 (52.1%)	561 (45.4%)
Difficulty bathing or dressing	Yes	242 (39.2%)	162 (26.1%)	404 (32.7%)
	No	376 (60.8%)	456 (73.8%)	832 (67.3%)
SF‐36 mental health score^c^	Mean ± SD	47.7 ± 10.3	50.3 ± 9.5	49.0 ± 9.9
SF‐36 physical health score^c^	Mean ± SD	43.1 ± 9.4	44.5 ± 8.8	43.8 ± 9.1
Comorbid conditions				
Diabetes	Yes	167 (27.0%)	142 (23.0%)	309 (25.0%)
	No	451 (73.0%)	476 (77.0%)	927 (75.0%)
Hypertension	Yes	530 (85.8%)	565 (91.4%)	1095 (88.6%)
	No	88 (14.2%)	53 (8.6%)	141 (11.4%)
Ischemic heart disease	Yes	429 (69.4%)	433 (70.1%)	862 (69.7%)
	No	189 (30.6%)	185 (29.9%)	374 (30.3%)
Stroke	Yes	178 (28.8%)	200 (32.4%)	378 (30.6%)
	No	440 (71.2%)	418 (67.6%)	858 (69.4%)
Number of prescription medications during the past 4 weeks	None	43 (7.0%)	50 (8.1%)	93 (7.5%)
	One	117 (18.9%)	167 (27.0%)	284 (23.0%)
	Two	150 (24.3%)	190 (30.7%)	340 (27.5%)
	Three	157 (25.4%)	155 (25.1%)	312 (25.2%)
	Four/more	151 (24.4%)	56 (9.1%)	207 (16.8%)
Duration illness (in years)	Mean (SD)	5.50 ± 4.36	3.31 ± 4.28	4.40 ± 4.56
Number of hospital admissions in last 12 months prior to last year of life	Median (IQR)	2 (1–5)	0 (0–1)	1 (0–3)
LOS in last 12 months prior to last year of life (in days)	Median (IQR)	17 (3–38)	0 (0–3)	3 (0–21.5)

Abbreviation: LOS, length of hospital stay.

^a^ Descriptive statistics are presented as mean ± standard deviation or median with interquartile range for continuous variables and frequency (%) for categorical variables.

^b^ DSSI, Duke Social Support Index, with the social interaction subscale score ranging from 4 to 12, with higher scores indicative of increased social interaction [30].

^c^ SF‐36 mental and physical component scores are standardised for Australian women of similar age.

### 3.2 Model Development

In univariate logistic regression analysis, all candidate predictors showed associations with the outcome, with *p*‐values < 0.25 (Table S1 in the Supporting Information). The Lasso regression process then selected 13 variables (Table S2 in the Supporting Information). Applying multivariable binary logistic regression and further model simplification, the final candidate predictors for model development were determined to be smoking, being underweight (BMI < 22 kg/m^2^), requiring regular help with daily tasks, being supplied with four or more medications in the past 4 weeks, the duration of illness, and the number of hospital admissions in the past 12 months prior to the last year of life (Tables S2 and S3 in the Supporting Information). The final prediction model was presented as a regression model equation, including the shrunken regression coefficients and the intercept.

The linear predictor (lp) is calculated as:

lp = −0.47 + 0.70* being a smoker +0.43* underweight BMI + 0.63* Regularly needing help with daily tasks +0.72* supplied four or more medications +0.10* duration of illness +0.28 * number of hospital admissions in the last year prior to the last year of life.

Consequently, the risk of 1‐year all‐cause mortality for each patient can be calculated as:

$$\begin{array}{c} P\;\left(1‐\text{year}\;\mathrm{all}‐\text{cause mortality}\right)=\exp^{\left(\mathrm{lp}\right)}/\left(1+\exp^{\left(\mathrm{lp}\right)}\right). \end{array}$$

To demonstrate the model's applicability and accessibility, a simple web‐based risk calculator was developed using the “Shiny” package. By entering individual patient characteristics, such as smoking status, BMI, need for daily assistance, duration of illness, and hospital admissions, the calculator automatically computes the predicted probability of 1‐year all‐cause mortality and categorises the risk. For example, consider an elderly woman with COPD for 9 years. She regularly needed assistance with daily tasks due to her long‐term illness, disability, or frailty. This patient had been receiving four or more medications, excluding over‐the‐counter medications, in the past 4 weeks and had one hospital admission in the past 12 months. Entering this patient's information into the web‐based calculator yielded an estimated probability of 1‐year all‐cause mortality of 88.7%. The calculator is accessible at [https://begash.shinyapps.io/COPD‐risk‐calculator/].

### 3.3 Performance of the Prediction Model

**FIGURE 2:**
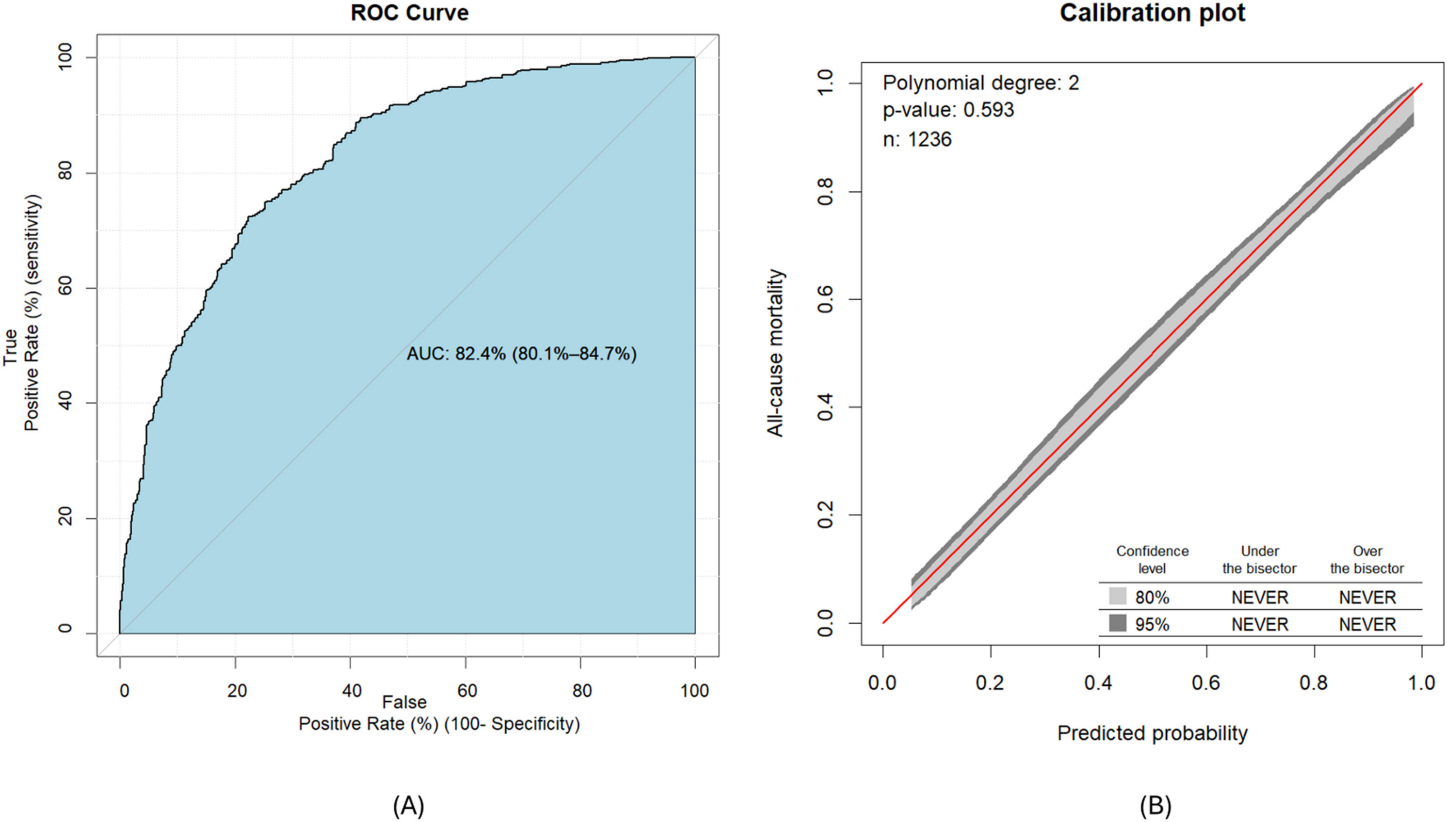
AUROC of the model demonstrating the discrimination performance of the model (A) and the calibration plot of the model, illustrating the agreement between predicted and observed probabilities (B).

**FIGURE 3:**
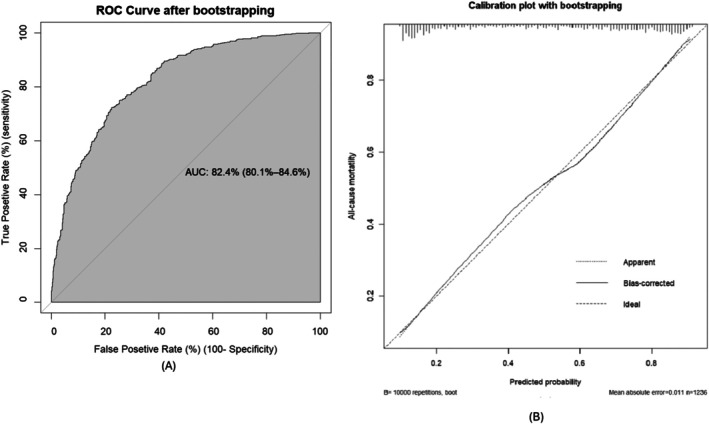
AUROC of the model during 10,000 rounds of bootstrap internal validation (A) and calibration plot of the model during 10,000 rounds of bootstrap internal validation (B), indicating minimal overfitting.

We assessed discrimination using AUROC; the final model with original beta coefficients achieved an AUROC of 82.4% (95% CI: 80.1%–84.7%) (Figure 2A). The developed model had a calibration plot *p*‐value of 0.593, which was greater than 0.05, and the 95% confidence intervals closely followed the diagonal line, indicating that the model was well calibrated (Figure 2B). Internal validation of the model was performed using the bootstrapping technique, and after 10,000 bootstrap replicates, the model achieved an AUROC of 82.4% (95% CI: 80.1%–84.6%) (Figure 3A). The model calibration was excellent, with a mean absolute error of 0.011 and a mean squared error of 0.00018 (Figure 3B).

We classified the patients into high‐ and low‐risk groups using an optimal cutoff for the predicted probability. This cutoff was selected by maximising the sum of the model's sensitivity and specificity to minimise false positives, which are unavoidable [31]. This approach determined the optimal cutoff probability to be 0.566. Using this threshold, 585 patients were classified as high risk, and 651 patients were classified as having a low risk of death. The model's sensitivity, specificity, and overall true prediction accuracy for all‐cause mortality using this cutoff were 72.3%, 77.7%, and 75.0%, respectively (Table 2). The sensitivity, specificity, positive predictive value, and negative predictive value of the model were also determined at different cutoff probabilities (Table S4 in the Supporting Information).

**TABLE 2:** Risk classification of the model among women with COPD from the 1921–1926 ALSWH cohort.

Predicted risk	Risk category	Women with COPD	Died	Sensitivity	Specificity	PPV	NPV
< 56.6%	Low risk	651 (52.7%)	171 (26.3%)	72.3%	77.7%	76.4%	73.7%
≥ 56.6%	High risk	585 (47.3%)	447 (83.2%)				

Abbreviations: NPV, negative predictive value; PPV, positive predictive value.

### 3.4 Clinical Usefulness of the Model

**FIGURE 4:**
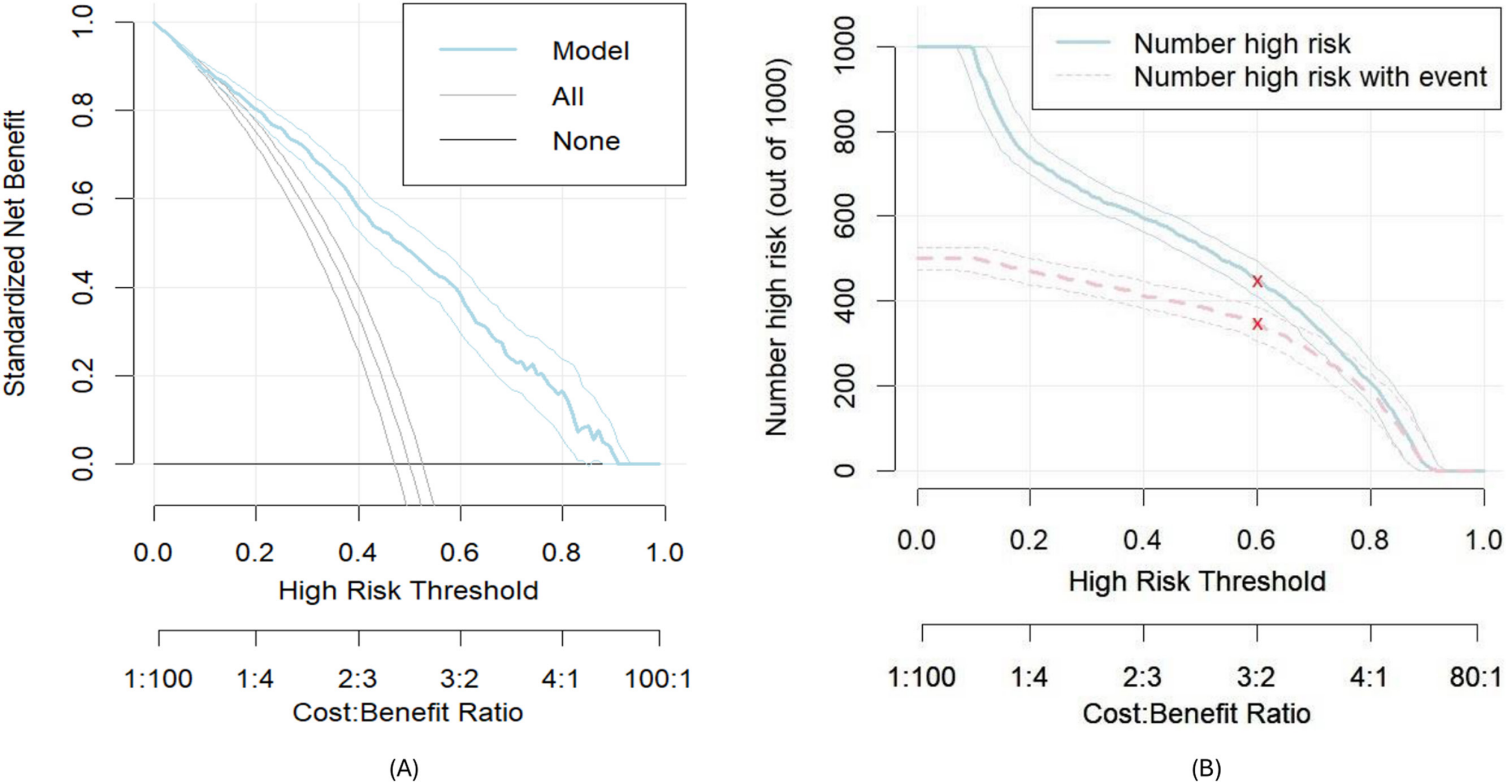
Decision curve analysis (A) and clinical impact plot (B) of the model demonstrating its net clinical benefit. At a 60% optimal risk threshold, applying the model to 1000 women with COPD classified 425 as high risk, of which 375 were true positives.

To explore the implications of using the model in clinical practice, we conducted a decision curve analysis to simulate outcomes if referring high‐risk patients to palliative care was based on various cutoff points from the model. The results are shown in Figure 4A,B. The analysis indicated that the model‐based referral approach provided a significantly better net benefit than not referring any patient. For an illustrative threshold representing a 50% risk of all‐cause mortality, using this model would offer a net benefit of 50% compared to an intervention in which no patients are referred to palliative care. In contrast, an intervention that refers all patients results in only an 8% net benefit compared with the refer‐none strategy, which has a net benefit of zero (Figure 4A). Thus, the model demonstrated a 42% net benefit over the strategy of referring all patients, highlighting its clinical importance. Additionally, a clinical impact plot was generated as part of a decision curve analysis to estimate the number of patients who would be classified as high‐risk at each risk threshold and to visualise the corresponding proportion of patients who would die (true positives). This analysis indicated that the model provided additional clinical value (Figure 4B).

## 4 Discussion

We developed a robust prediction model for all‐cause mortality among women with COPD, using data from the ALSWH old cohort. Our model was designed to identify high‐risk individuals with COPD who may benefit from proactive palliative care by utilising socio‐demographic, behavioural, and clinical characteristics. The final model included six key predictors: past or current smoking, underweight body mass index, regular need for assistance with daily activities, prescription of four or more medications, duration of illness, and number of hospital admissions. This model achieved good predictive performance, with an AUROC of 0.82(95% CI 0.80, 0.85), indicating a high level of accuracy in distinguishing between patients who will and will not die within 1 year.

The model incorporated six key prognostic determinants that were independently associated with a high risk of mortality. These predictors capture critical aspects of disease severity and progression, including ongoing respiratory damage and susceptibility to exacerbations from smoking; nutritional depletion from underweight BMI; multimorbidity, treatment burden, and complexity from multiple prescribed medications; chronic disease progression from longer illness duration; and severe exacerbations and health deteriorations from frequent hospital admissions [24, 32, 33]. Additionally, the model accounts for more severe disease progression where functional decline significantly affects daily living [24, 32]. By incorporating these variables, the model enables a comprehensive assessment to identify high‐risk patients, facilitating timely interventions and referral to palliative care in the last year of life [32].

Our model demonstrated better predictive performance than existing indices, such as ADO (AUC: 0.70) [23, 24, 34], BODE (AUC: 0.68) [23, 24], and SAFE (AUC: 0.64) [23, 24], in predicting mortality among individuals with COPD, suggesting its potential effectiveness in identifying individuals with COPD who would benefit from proactive palliative care. This finding may be attributed to the distinct socio‐demographic characteristics of our study participants, who were exclusively females aged 70 years or older. In addition, the model demonstrated excellent calibration with a *p*‐value of 0.593, a mean absolute error of 0.011, and a mean squared error of 0.00018. These results indicate that the predicted mortality probabilities of the model closely aligned with the observed mortality rates, highlighting its robust accuracy. In contrast, a statistically significant (*p* < 0.05) result suggests discrepancies between the predicted and actual outcomes, implying a poorer calibration.

According to our model, the risk of all‐cause mortality increased as the probability value increased. Using the identified optimal cutoff probability, patients in the high‐risk category had a significantly higher risk of mortality, with an odds ratio of 9.09, compared to those in the low‐risk category. Healthcare providers can use different cutoff points depending on resource availability. If providers value sensitivity and specificity equally, the model's cutoff value of 0.566 maximises both. However, providers may choose different cutoff points based on the relative importance of false positives and false negatives in their specific contexts.

False positives and false negatives carry distinct and significant implications in clinical practice [31, 35]. False positives can result in unnecessary palliative care referrals, potentially causing emotional distress for patients and their families while imposing additional burdens on already constrained healthcare resources [31, 35]. Conversely, false negatives may result in missed opportunities for timely palliative care, denying patients access to essential symptom management and potentially compromising their quality of life in their final months [31, 35]. In many clinical contexts, prioritising sensitivity by accepting a higher rate of false positives may be advantageous, particularly when the consequences of failing to identify high‐risk patients outweigh the costs of over‐referral [31, 35]. This trade‐off highlights the critical need to tailor cutoff probabilities to the specific priorities and resource constraints of individual healthcare settings [31, 35].

Although the performance of our model in terms of discrimination and calibration was evaluated, its clinical usefulness needs to be further assessed to promote its applicability in the clinical setting. Therefore, we conducted a decision curve analysis to evaluate the model's clinical usefulness, which indicated that the model‐based referral approach provided a significantly better net benefit than referring all or no patients across a wide range of threshold probabilities. This suggests that the model has important clinical and public health value, and healthcare providers can effectively use the developed risk score calculator to identify high‐risk individuals with COPD for proactive palliative care referral, following careful external validation.

### 4.1 Strength and Limitations

The model was developed using readily available, noninvasive, and cost‐effective socio‐demographic, behavioural, and clinical factors, making it a simple risk assessment tool that healthcare providers can employ in various settings. It can be easily integrated into web‐based applications, aiding in the identification of individuals with COPD at high risk for timely palliative care interventions. The potential advantages of such technological integration include streamlined workflows, real‐time risk evaluations, and improved patient outcomes. However, the model has limitations: it requires external validation to assess its performance in different settings, and the study focused on a specific age group of women, which may limit its generalisability. In addition, the case definition of COPD relied on participants' self‐reported diagnosis or ICD codes, which introduce a potential source of misclassification bias. Although our model did not incorporate determinants such as airflow obstruction as measured by forced expiratory volume in 1 s, previous models that incorporated this parameter performed worse than our current model [23, 24].

Even though mortality is not the only factor considered in palliative care assessment, models such as this can be helpful tools for healthcare providers and decision makers in identifying patients who may benefit from referral to palliative care. By adjusting the sensitivity and specificity of these models, the burden on healthcare professionals can be reduced by focusing on a manageable subset of patients for palliative care based on available resources. These models can also help prioritise patient needs by predicting the mortality risk within a specific timeframe. However, it is important to note that these models should not be used alone, as algorithm‐generated mortality predictions do not fully capture the complex nature of palliative care need. The value of palliative care extends beyond assessing the risk of death.

### 4.2 Potential Applications

Reliable prognostic information on patients' 1‐year mortality risk could assist healthcare providers in making decisions regarding when to initiate advance care planning discussions, facilitating proactive palliative care and planning. Moreover, integrating risk score calculators into educational programs on advance care planning could enhance healthcare providers' understanding and use of proactive palliative care strategies. Additionally, the application of this tool in clinical practice may improve patient outcomes by ensuring that high‐risk patients receive the necessary palliative care interventions in a timely manner. Future research should focus on validating the model's performance in diverse populations and settings to ensure its robustness and adaptability in clinical practice, especially to verify whether this model can be extended to males.

In conclusion, we developed a predictive model for 1‐year all‐cause mortality among women with COPD using data from the older ALSWH cohort. The model included six key predictors: smoking status, underweight BMI, regular need for assistance with daily activities, prescription of four or more medications, duration of illness, and number of hospital admissions. The model demonstrated good predictive performance, with good discriminative ability, calibration, and usefulness. This model can be used to stratify women with COPD into different risk groups, enabling targeted palliative care intervention. For patients with similar population characteristics, this model can support healthcare providers in identifying high‐risk individuals and tailoring interventions accordingly. However, external validation of the developed model is necessary to confirm its performance and applicability across diverse clinical settings and to improve its generalisability.

## Author Contributions

**Begashaw Melaku Gebresillassie:** conceptualization (equal), data curation (lead), formal analysis (lead), investigation (lead), methodology (lead), software (lead), visualization (lead), writing – original draft (lead), writing – review and editing (lead). **John Attia:** conceptualization (equal), methodology (equal), project administration (equal), supervision (equal), validation (equal), writing – review and editing (equal). **Dominic Cavenagh:** data curation (supporting), formal analysis (supporting), methodology (supporting), project administration (equal), validation (equal). **Melissa L. Harris:** conceptualization (equal), methodology (equal), project administration (equal), supervision (equal), validation (equal), writing – review and editing (equal).

## Ethics Statement

All data for this study were obtained from the ALSWH, which was approved under their expression of interest process (EoI: A1390) and provided in a de‐identified form. This study has ongoing ethical clearance from the University of Newcastle and University of Queensland’s Human Research Ethics Committees. Ethical approval for the linkage of ALSWH data to the admitted patient data collection was received from each state and territory‐based committee. Prior to 2005, women provided written consent to participate in the ALSWH and for their data to be linked to de‐identified administrative health records. From 2005 onwards, an ‘opt‐out’ consent process was approved by the data custodians and relevant ethics committees for data linkage, with participants regularly reminded of this process. ALSWH participants who declined health record linkage were excluded from the data linkage requests.

## Conflicts of Interest

The authors declare no conflicts of interest.

## Supporting information


**Data S1** Supporting Information.

## Data Availability

The datasets supporting the findings of this study are available through the Australian Longitudinal Study on Women's Health; http://www.alswh.org.au/for‐researchers.
