# Supplementary material for: Development and Validation of a Risk Prediction Model to Identify Women With Chronic Obstructive Pulmonary Disease for Proactive Palliative Care
Source: Respirology. 2025 Feb 16;30(7):623–32. doi: 10.1111/resp.70005 (PMC12231770; doi:10.1111/resp.70005)
Supplement: Supplementary file 1 — Data S1 Supporting Information. [file RESP-30-623-s001.docx]

**SUPPORTING INFORMATION**

**Development and validation of a risk prediction model to identify women with chronic obstructive pulmonary disease for proactive palliative care**

Begashaw Melaku Gebresillassie^1,2,3^*, John Attia^1,3^, Dominic Cavenagh^2,3^, Melissa L. Harris^1,2,3^

^1^School of Medicine and Public Health, The University of Newcastle, Newcastle, New South Wales, Australia

^2^Centre for Women’s Health Research, University of Newcastle, Newcastle, Australia

^3^Hunter Medical Research Institute, Newcastle, New South Wales, Australia

*Corresponding author

Begashaw Melaku Gebresillassie

Email: [begashaw531@gmail.com](mailto:begashaw531@gmail.com) or [begashawmelaku.gebresillassie@uon.edu.au](mailto:begashawmelaku.gebresillassie@uon.edu.au)

Phone: +61490914058

**Details on chronic obstructive pulmonary disease (COPD) case ascertainment**

COPD was ascertained using the Australian Longitudinal Study on Women's Health (ALSWH) -developed Common Conditions from Multiple Sources (CCMS) datasets. These datasets developed from multiple sources including a) ALSWH survey data: COPD cases were based on participants' self-reported diagnosis or treatment of COPD. Additionally, free text comments were also searched for terms such as 'copd', 'coad', 'emphys', 'chronic obstruct', and 'chronic bronchitis'. b) Admitted Patient Data collections (APDC) and Emergency Department Data Collections: Admission and separation dates, as well as the primary and secondary diagnoses assigned by physicians, were coded using the International Statistical Classification of Diseases and Related Health Problems Australian Modification (ICD-AM) versions 9 and 10 (depending on the time frame of the data coverage). c) National Death Index (NDI): This provides information on all deaths occurring in Australia. Deaths related to COPD were recorded in a similar manner to the admitted patients hospital data, using ICD-AM codes. COPD cases were identified using the same methods as those used for the admitted patients hospital data. d) Pharmaceutical Benefits Scheme (PBS): This provides information on subsidised prescription medications for eligible Australian citizens, residents, and foreign visitors covered by a Reciprocal Health Care agreement. Prior to April 2012, only prescriptions that carried a government subsidy and were above the co-payment were recorded. From April 2012 onwards, all government-subsidised medications, both above and below the co-payment, were captured through the PBS database. COPD cases were identified using the World Health Organisation's Anatomical and Therapeutic Chemical Classification (ATC) codes. Medications typically indicated for the treatment of COPD were used to identify the COPD cases. e) Aged care assessments: These data were obtained from the Australian Institute of Health and Welfare, which extracted records for all ALSWH participants in the 1921-1926 cohort. Several sources were used to identify COPD records: the Aged Care Assessment Program (which assesses the care needs of older people and assists in accessing appropriate types of care), the Aged Care Funding Instrument (which assesses care needs as a basis for calculating and allocating funds to the aged care facility), and the National Screening and Assessment Form (NSAF). To identify COPD cases, the diagnosis code '1005' for chronic lower respiratory diseases, which includes emphysema and chronic obstructive airways disease, was used. This diagnosis was obtained through referrals to a general practitioner, geriatrician, or psycho-geriatrician, or through an assessor (with consent) accessing medical history information from a relevant doctor.

To be included as a COPD case, the COPD diagnosis needs to meet at least one of the following criteria: (a) reported once or more in admitted hospital data and NDI; or (b) reported once or more in aged care assessments and once or more in ALSWH, PBS, or admitted hospital data; (c) reported in two or more ALSWH surveys with breathing difficulties reported as 'often' twice or more (excluding asthma), and reported once or more in PBS, admitted hospital data, or aged care assessments; or (d) two or more medications prescribed within a 12-month period, reported in two separate calendar years (as it was assumed that COPD would require regular ongoing medications), or reported once and once or more in admitted hospital data or aged care assessments.

**Sample size calculation**

The sample size for the model development was calculated using the 'pmsampsize' package, which is based on recommendations by Riley et al ^1, 2^. Given the binary nature of the primary outcome, the parameter 'type' was set to "b". With approximately 20 candidate predictors proposed for the new model and a c-statistic of 0.82 from the previous study ^3^, the mortality prevalence was estimated at 20.7% ^4^. Employing the "pmsampsize" command and assuming an acceptable difference of 0.05 in apparent and adjusted R-squared, as well as a margin of error of 0.05 in intercept estimation, the minimum sample size required for the new model development was determined to be 746, with 7.72 events per predictor variable. This indicates that the sample size used for our predictive model development was adequately robust for the model development process.

**Table S1: Outlines a list of potential candidate predictors considered for model development.**

| **Candidate predictors** | **Categories** | **Univariable analysis** | |
| --- | --- | --- | --- |
|  |  | **COR (95%CI)** | **P-value** |
| Baseline age in 1996 | In years | 1.15(1.07-1.25) | <001 |
| Marital status | Partnered | 1 |  |
|  | Non-partnered | 0. 67(0.53-0.86) | 0.002 |
| Smoking status | Never smoked | 1 |  |
|  | Current/past-smoker | 1.96(1.56-2.46) | <001 |
| Social interaction according to Duke Social Support Index ^5^. | Mean ± SD | 0.94 (0.88-1.01) | 0.096 |
| Body mass index (BMI) categorised according to Nutritional Intervention Manual for Profession Caring for Older people which is the standard for measurement among older adults ^6^. | Healthy weight [22-27 kg/m²] | 1 |  |
|  | Underweight [<22 kg/m²] | 1.79(1.36-2.35) | <001 |
|  | Overweight/ obese [>27 kg/m²] | 1.17(0.89-1.53) | 0.253 |
| Private health insurance | Yes | 1 |  |
|  | No | 1.25(0.99-1.58) | 0.057 |
| Compared to one year ago, how would you rate your health in general now | Better | 1 |  |
|  | Same/worse | 1.35(0. 93-1.97) | 0.110 |
| Do you regularly need help with daily tasks because of long-term illness, disability or frailty (eg personal care, getting around, preparing meals etc)? | Yes | 2.38(1.85-3.08) | <001 |
|  | No | 1 |  |
| During a typical day, does your health now limit you in these activities? If so, how much? Lifting or carrying groceries | Yes | 1.58(1.20-2.08) | 0.001 |
|  | No | 1 |  |
| During a typical day, does your health now limit you in these activities? If so, how much? Climbing one flight of stairs | Yes | 1.55(1.21- 1.98) | 0.001 |
|  | No | 1 |  |
| During a typical day, does your health now limit you in these activities? If so, how much? Bending, kneeling or stooping | Yes | 1.30(0.98-1.72) | 0.072 |
|  | No | 1 |  |
| During a typical day, does your health now limit you in these activities? If so, how much? Walking half a kilometre | Yes | 1.31(1.03-1.68) | 0.029 |
|  | No | 1 |  |
| During a typical day, does your health now limit you in these activities? If so, how much? Walking 100 metres | Yes | 1.72(1.37-2.16) | <0.001 |
|  | No | 1 |  |
| During a typical day, does your health now limit you in these activities? If so, how much? Bathing or dressing yourself | Yes | 1.81(1.42-2.31) | <0.001 |
|  | No | 1 |  |
| How many different types of medication (eg. tablets or medicine) that were prescribed or recommended by a doctor have you used during the last 4 weeks? | None | 1 |  |
|  | One | 0.81(0.51-1.30) | 0.394 |
|  | Two | 0.92(0.58-1.45) | 0.716 |
|  | Three | 1.18(0.74-1.87) | 0.490 |
|  | Four and more | 3.13(1.88-5.22) | <0.001 |
| Comorbid Diabetes conditions | Yes | 1.24(0.96- 1.61) | 0.101 |
|  | No | 1 |  |
| Dementia | Yes | 0.67(0 .53-0 .85) | 0.001 |
|  | No | 1 |  |
| Hypertension | Yes | 0.56(0.39-0 .81) | 0.002 |
|  | No | 1 |  |
| Stroke | Yes | 0.84(0 .66- 1.08) | 0.175 |
|  | No | 1 |  |
| Duration of illness in years calculated from the date first diagnosis recorded | Mean ± SD | 1.12(1.09 0 1.15) | <0.001 |
| SF-36 mental health score^‡^ | Mean ± SD | 0.97(0.96-0.98) | <0.001 |
| SF-36 physical health score^‡^ | Mean ± SD |  |  |
| Number of hospital admissions in last 12 months prior to last year of life | Median (IQR) | 1.32(1.27 – 1.36) | <0.001 |
| Perceives health as excellent | Yes | 1 |  |
|  | No | 2.06(1.64-2.60) | <0.001 |
| Compared to one year ago, health now | Better | 1 |  |
|  | Same/worse | 1.35(0.93-1.97) | 0.110 |

Abbreviation: COR: Crude Odds Ratio; ^‡^SF-36 mental and physical components scores are standardised for Australian women of similar age.

**Table S2:** Prognostic determinants of one-year all-cause mortality among women with COPD from the 1921-26 ALSWH cohort, (n=1236)

| **Predictors selected by Lasso** | **Died** | **Alive** | **Multivariable analysis for Lasso-selected variables** | | **Multivariable analysis for refined variables** | |
| --- | --- | --- | --- | --- | --- | --- |
|  |  |  | **AOR (95%CI)** | **P-value** | **AOR (95%CI)** | **P-value** |
| Smoking status |  |  |  |  |  |  |
| Never smoked | 218 | 319 | 1 |  | 1 |  |
| Current/ex-smoker | 400 | 299 | 1.99(1.50-2.63) | <0.001* | 2.01(1.53-2.66) | <0.001* |
| Body mass index |  |  |  |  |  |  |
| Healthy | 212 | 266 | 1 |  | 1 |  |
| Underweight | 224 | 157 | 1.54 (1.11-2.16) | 0.010* | 1.54(1.11-2.14) | 0.010* |
| Overweight/obese | 182 | 195 | 0.88(0.63-1.23) | 0.449 | 0.88(0.63-1.23) | 0.460 |
| Private health insurance |  |  |  |  |  |  |
| Yes | 202 | 234 | 1 |  |  |  |
| No | 416 | 384 | 1.27(0.95-1.70) | 0.099 |  |  |
| Regularly need help with daily tasks |  |  |  |  |  |  |
| Yes | 233 | 125 | 1.70(1.19-2.429) | 0.003* | 1.88(1.38-2.56) | <0.001* |
| No | 385 | 493 | 1 |  | 1 |  |
| Difficulty lifting and carrying |  |  |  |  |  |  |
| Yes | 510 | 463 | 1.08(0.75-1.57) | 0.663 |  |  |
| No | 108 | 155 | 1 |  |  |  |
| Difficulty walking 100 meters |  |  |  |  |  |  |
| Yes | 379 | 296 | 0.95(0.69-1.31) | 0.765 |  |  |
| No | 239 | 322 | 1 |  |  |  |
| Difficulty bathing or dressing |  |  |  |  |  |  |
| Yes | 242 | 162 | 1.09(0.77-1.55) | 0.622 |  |  |
| No | 376 | 456 |  |  |  |  |
| SF-36 mental health score ^d^ |  |  |  |  |  |  |
| Mean ± SD | 47.7 ± 10.3 | 50.3 ± 9.5 | 1.00(0.99-1.02) | 0.738 |  |  |
| Number of prescription medications during the past 4 weeks |  |  |  |  |  |  |
| None | 43 | 50 | 1 |  | 1 |  |
| One | 117 | 167 | 0.66(0.37-1.16) | 0.151 | 0.67(0.38-1.18) | 0.166 |
| Two | 150 | 190 | 0.71(0.40-1.26) | 0.247 | 0.76(0.44-1.31) | 0.323 |
| Three | 157 | 155 | 0.82(0.46-1.46) | 0.501 | 0.87(0.50-1.53) | 0.640 |
| Four/more | 151 | 56 | 1.87(0.99-3.54) | 0.053 | 2.05(1.11-3.78) | 0.021* |
| Duration of illness (in years) |  |  |  |  |  |  |
| Mean (SD) | 5.5 ± 4.4 | 3.3 ± 4.3 | 1.10(1.07 – 1.14) | <0.001* | 1.10(1.07-1.14) | <0.001* |
| Number of hospital admission in last 12 months |  |  |  |  |  |  |
| Median (IQR) | 2 (1 – 5) | 0 (0 – 1) | 1.32(1.27-1.37) | <0.001* | 1.32(1.27-1.37) | <0.001* |
| Perceives health as excellent |  |  |  |  |  |  |
| Yes | 207 | 315 | 1 |  |  |  |
| No | 411 | 303 | 1.28(0.92-1.78) | 0.148 |  |  |
| Compared to one year ago, health now |  |  |  |  |  |  |
| Better | 54 | 71 | 1 |  |  |  |
| Same/worse | 564 | 547 | 1.14(0.73-1.80) | 0.565 |  |  |

*AOR:* *Adjusted Odds Ratio; *P<0.05*

**Table S3:** Prognostic determinants of one-year all-cause mortality utilised to develop the model.

| **Predictors** | **Regression coefficients** | **AOR (95%CI)** | **P-value** |
| --- | --- | --- | --- |
| Being current/past smoker | 0.70 | 2.01(1.53-2.66) | <0.001 |
| Underweight BMI | 0.43 | 1.54(1.11-2.14) | 0.010 |
| Regularly need help with daily tasks (yes vs no) | 0.63 | 1.88(1.38-2.56) | <0.001 |
| Supplied 4 or more medications during the past 4 weeks (yes vs no) | 0.72 | 2.05(1.12-3.78) | 0.021 |
| Duration of illness (per 1 year increase) | 0.10 | 1.10(1.07 – 1.14) | <0.001 |
| Log number of hospital admission in the past 12 months (per 1 log hospital admission increase) | 0.28 | 1.32(1.27-1.37) | <0.001 |
| Constant | -0.47 | | |

Table S4: Performance of the model at different cutoff points.

| Cutoff point | Sensitivity (%) | Specificity (%) | PPV (%) | NPV (%) |
| --- | --- | --- | --- | --- |
| 0.400 | 84.9 | 62.3 | 69.3 | 80.5 |
| 0.500 | 77.2 | 70.4 | 72.3 | 75.5 |
| 0.566 | 72.3 | 77.7 | 76.4 | 73.7 |
| 0.600 | 67.0 | 80.1 | 77.1 | 70.8 |
| 0.700 | 51.5 | 88.8 | 82.2 | 64.7 |

Abbreviations: PPV, Positive Predictive Value, NPV, Negative Predictive Value

**References**

1 Riley RD, Ensor J, Snell KI, Harrell FE, Martin GP, Reitsma JB, Moons KG, Collins G, Van Smeden M. Calculating the sample size required for developing a clinical prediction model. Bmj. 2020; **368**.

2 Riley RD, Snell KI, Ensor J, Burke DL, Harrell Jr FE, Moons KG, Collins GS. Minimum sample size for developing a multivariable prediction model: PART II‐binary and time‐to‐event outcomes. Statistics in medicine. 2019; **38**: 1276-96.

3 Duenk R, Verhagen C, Bronkhorst E, Djamin R, Bosman G, Lammers E, Dekhuijzen P, Vissers K, Engels Y, Heijdra Y. Development of the ProPal-COPD tool to identify patients with COPD for proactive palliative care. International journal of chronic obstructive pulmonary disease. 2017: 2121-8.

4 Cranston JM, NGUYEN AM, Crockett AJ. The relative survival of COPD patients on long‐term oxygen therapy in Australia: A comparative study. Respirology. 2004; **9**: 237-42.

5 ALSWH. ALSWH Data Dictionary Supplement Section: Duke Social Support Index (DSSI). <https://alswh.org.au/wp-content/uploads/2020/08/DDSSection2.7DSSI.pdf>.

6 Blackburn G, Dwyer J, Wellman N. Nutrition interventions manual for professionals caring for older Americans. Washington, DC: The Nutrition Screening Initiative. 1992.
